# Sphingosine-1-Phosphate Receptor 3 Mediates Sphingosine-1-Phosphate Induced Release of Weibel-Palade Bodies from Endothelial Cells

**DOI:** 10.1371/journal.pone.0091346

**Published:** 2014-03-14

**Authors:** Kathinka W. E. M. van Hooren, Léon J. A. Spijkers, Dorothee van Breevoort, Mar Fernandez-Borja, Ruben Bierings, Jaap D. van Buul, Astrid E. Alewijnse, Stephan L. M. Peters, Jan Voorberg

**Affiliations:** 1 Department of Plasma Proteins, Sanquin-AMC Landsteiner Laboratory, Amsterdam, The Netherlands; 2 Department of Pharmacology and Pharmacotherapy, Academic Medical Center, Amsterdam, The Netherlands; 3 Department of Molecular Cell Biology, Sanquin-AMC Landsteiner Laboratory, Amsterdam, The Netherlands; Institut National de la Santé et de la Recherche Médicale, France

## Abstract

Sphingosine-1-phosphate (S1P) is an agonist for five distinct G-protein coupled receptors, that is released by platelets, mast cells, erythrocytes and endothelial cells. S1P promotes endothelial cell barrier function and induces release of endothelial cell-specific storage-organelles designated Weibel-Palade bodies (WPBs). S1P-mediated enhancement of endothelial cell barrier function is dependent on S1P receptor 1 (S1PR1) mediated signaling events that result in the activation of the small GTPase Rac1. Recently, we have reported that Rac1 regulates epinephrine-induced WPB exocytosis following its activation by phosphatidylinositol-3,4,5-triphosphate-dependent Rac exchange factor 1 (PREX1). S1P has also been described to induce WPB exocytosis. Here, we confirm that S1P induces release of WPBs using von Willebrand factor (VWF) as a marker. Using siRNA mediated knockdown of gene expression we show that S1PR1 is not involved in S1P-mediated release of WPBs. In contrast depletion of the S1PR3 greatly reduced S1P-induced release of VWF. S1P-mediated enhancement of endothelial barrier function was not affected by S1PR3-depletion whereas it was greatly impaired in cells lacking S1PR1. The Rho kinase inhibitor Y27632 completely abrogated S1P-mediated release of VWF. Also, the calcium chelator BAPTA-AM significantly reduced S1P-induced release of VWF. Our findings indicate that S1P-induced release of haemostatic, inflammatory and angiogenic components stored within WPBs depends on the S1PR3.

## Introduction

Sphingosine 1-phosphate (S1P) is a naturally occurring lysophospholipid that plays a crucial role in maintaining vascular homeostasis [1], [2]. S1P production is tightly regulated by two sphingosine kinases and a number of degradative enzymes that include two S1P-phosphatases, an S1P-lyase and lysophospholipid phosphatases [1]. S1P levels in lymphatic tissues are generally low and there is strong evidence that lymphocyte egress from these sites is controlled by the S1P/S1P1 receptor axis [3], [4]. Circulating S1P levels mainly originate from endothelium, platelets and erythrocytes and vary between 0.4–1.5 μM in blood where it is bound to albumin and other plasma proteins [1], [5], [6]. S1P exerts its extracellular effects through high-affinity binding to G protein-coupled receptors sphingosine-1-phosphate receptors 1–5 (S1PR1-5) [1], [7]. In mammals, S1PR1, S1PR2, and S1PR3 are ubiquitously expressed, whereas S1PR4 expression is restricted to lymphoid tissues and lung, and S1PR5 to brain and skin[8]. S1PR1, S1PR2 and S1PR3 are the major S1P receptors in the cardiovascular system [9]. It is well-established that S1P promotes the barrier function of endothelial cells [10]. A crucial role for the S1PR1 and downstream signaling pathways resulting in activation of Rac1 have been defined in follow-up studies [11], [12]. Apart from its effect on endothelial cell barrier function S1P has been shown to induce the exocytosis of endothelial cell specific storage-organelles designated Weibel-Palade bodies (WPBs) [13], [14]. WPBs are rod-shaped organelles that store von Willebrand factor (VWF), a multimeric adhesive glycoprotein crucial for platelet plug formation as well as the leukocyte receptor P-selectin [15], [16]. Also, CD63 a co-receptor for P-selectin is stored within these organelles firmly linking WPB exocytosis to leukocyte extravasation [17], [18]. Vaso-active components like endothelin and calcitonin-gene related peptide as well as the angiogenic mediators angiopoietin-2 and insulin-like growth factor binding protein 7 (IGFBP7) are also stored in WPBs [19]–[22]. A number of other bioactive compounds that include chemoattractants like interleukin 6 and 8 and eotaxin-3 have also been localized to these organelles [23]–[25].

Release of WPB-content in the vessel lumen occurs following stimulation with agonists such as thrombin, histamine and epinephrine [26]–[28]. The vasopressin analogue desmopressin has been shown to mediate WPB release following stimulation of vasopressin 2 receptor that is expressed on lung endothelial cells. S1P has also been described to trigger WPB secretion in a concentration dependent manner [14]. As yet the receptor involved in S1P-induced release of WPBs has not yet been defined [14]. In this study we explored which S1P receptor regulates WPB secretion; we also determined which downstream signaling pathways contribute to the S1P induced release of these organelles.

## Methods

### Reagents and antibodies

All culture media (except EBM-2 and EGM-2), L-cysteine, fibronectin, trypsin, penicillin, and streptomycin were from Invitrogen (Breda, the Netherlands). EBM-2 and EGM-2 were from Lonza (Verviers, Belgium). Epinephrine, thrombin, forskolin, 3-isobutyl-1-methylxanthine (IBMX), LY294002, Y27632, BAPTA-AM, Endothelial Cell Growth Supplement (ECGS), and anti-α-tubulin monoclonal antibody (DM1A) were from Sigma-Aldrich Chemie (Steinheim, Germany). S1P was from Avanti Polar Lipids (Alabaster, Alabama, USA). Anti-β-catenin rabbit polyclonal antibody (sc-7199) was from Santa Cruz Biotechnology (Santa Cruz, California, USA). Chemiluminescence blotting substrate and Complete Protease Inhibitor Cocktail Tablets were from Roche Diagnostics (Mannheim, Germany). All chemicals used were of analytical grade. Anti-VWF monoclonal antibody CLB-RAg20 has been described previously [29]. Enzyme-linked immunosorbent assays (ELISA) for VWF and VWF propeptide have been described previously [30].

### Cell culture

Human umbilical vein endothelial cells (HUVECs) were obtained from Promocell (Heidelberg, Germany) and cultured in EGM-2 medium enriched with 10% fetal calf serum. Stimulation of endothelial cells with secretagogues was performed in the following manner: HUVECs, grown in 6-well plates, were washed two times with serum-free medium (SF medium: M199 and RPMI1640 (1∶1); 0.3 mg/ml L-glutamine; 100 units/ml penicillin; 100 mg/l streptomycin). Cells were pre-incubated in SF medium for 1 hour and subsequently SF medium with agonist was added. The agonists used were thrombin (1 U/ml); a mixture of 10 μM forskolin and 100 μM IBMX; or 1 μM S1P.

### siRNA

All siRNAs were purchased from Dharmacon (Thermo Fisher Scientific, Bremen, Germany). For siRNA mediated knock-down of S1PR1, ON-TARGETplus SMARTpool L-003655-00 Human EDG1 (1901) was used. For siRNA mediated knock-down of S1PR3, ON-TARGETplus SMARTpool L-005208-00 Human EDG3 (1903) was used. SiGENOME Non-Targeting siRNA Pool #1 (D-001206-13-05) was used as a control in these experiments. SiRNA (20 nM) was delivered to HUVECs by transfection using Interferin (PolyPlus, Westburg, Leusden, the Netherlands) according to the manufacturer's instructions essentially as described previously [31], [32]. Transfected HUVECs were grown on gelatin-coated multiwell-6 plates for 72 hours before stimulation. To confirm knockdown, total lysates were separated on NuPAGE 4–12% Bis-Tris Gels and transferred on nitrocellulose membrane (Invitrogen, Breda, the Netherlands). Membranes were probed with an anti-EDG1/S1PR1 rabbit polyclonal antibody (AP01197PU-N, Acris, Herford, Germany) or an anti-EDG3/S1PR3 rabbit polyclonal antibody (LS-B2155, Lifespan Biosciences, Seattle, WA, USA) and proteins were visualized by LI-COR Odyssey (Lincoln, NE, USA). Staining of the membranes with α-tubulin was used as a control for equal sample loading.

### Electric Cell-substrate Impedance Sensing

For Electrical Cell Substrate Impedance Sensing (ECIS)-based cell spreading experiments, golden ECIS electrodes (8W10E; Applied Biophysics, Troy, NY, USA) were treated with 10 μM L-cysteine for 15 minutes and subsequently coated with 5 μg/ml fibronectin in 0.9% NaCl for 1 hour at 37°C. Next, HUVECs, transfected with the indicated constructs were seeded at a concentration of 100,000 cells per array (surface area 0.8 cm^2^) in EGM-18 medium. The arrays are connected to a phase-sensitive lock-in amplifier that allows continuous recordings of the electrical resistance of the monolayers, which was on average between 1 and 1.5×10^3^ Ω. Basal electrical resistance of the endothelial monolayers under resting conditions was continuously monitored at 37°C at 5% CO_2_ with the electric cell-substrate impedance sensing (ECIS) model 100 controller (BioPhysics, Troy, NY). The impedance, as a measure of cell spreading and adherence was recorded for 18 hours before onset of the actual stimulation experiments. During stimulation experiments cells were pre-incubated in SF medium for 1 hour and subsequently SF medium containing 1 μM S1P was added.

## Results

**Figure 1:**
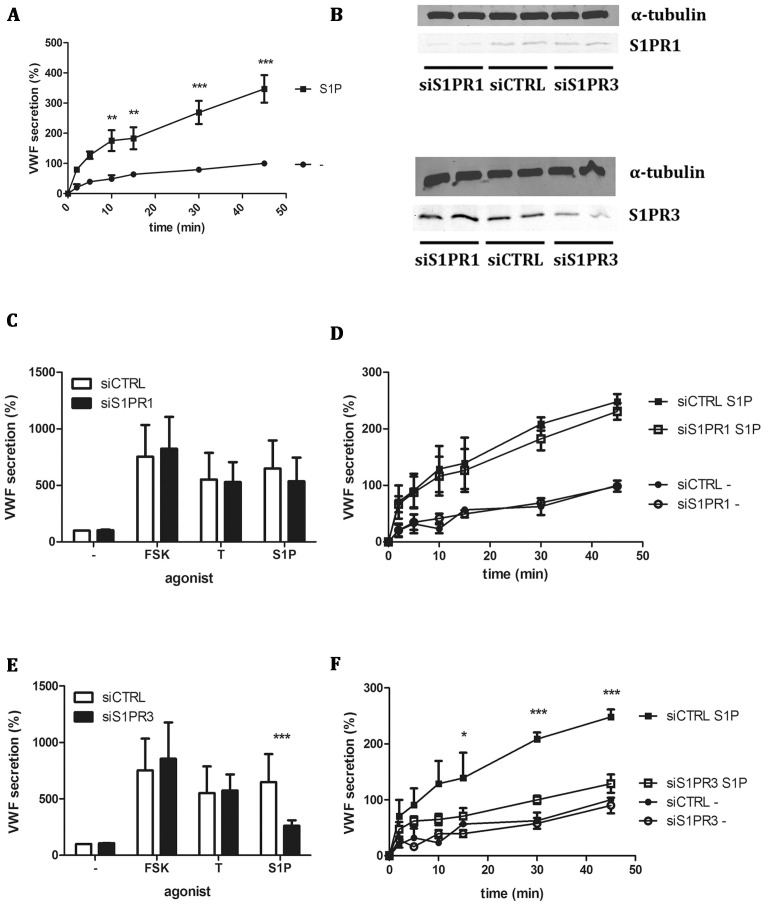
Downregulation of S1PR3 expression reduces WPB release. (A) HUVECs were incubated for the indicated time points with 1 μM S1P or SF medium. (B) HUVECs were transfected with a control siRNA SMARTpool (siCTRL) or a siRNA SMARTpool targeting S1PR1 (siS1PR1) or S1PR3 (siS1PR3). Western blot analysis at 72 hours post-transfection showed downregulation of S1PR1 expression or S1PR3 expression. Levels of α-tubulin are shown as a protein loading control. (C,E) siCTRL siS1PR1 and siS1PR3 treated HUVECs were incubated for 45 minutes with 1 μM S1P, 10 μM forskolin (FSK) and 100 μM IBMX, 1 U/ml thrombin (T) or SF medium alone (-). (D,F) siCTRL siS1PR1 and siS1PR3 treated HUVECs were incubated for indicated time points with 1 μM S1P. The amount of VWF secreted in the medium was measured by ELISA. Total amount of VWF secreted is displayed in percentages where the amount of VWF secreted by unstimulated cells t = 45 transfected with siCTRL is set to 100%. Four independent experiments were performed.Statistical significance was assessed by 2-way ANOVA followed by Bonferroni post-hoc test for selected comparison (***P<0.001; **P<0.01 and *P<0.05).

The sphingolipid S1P has been shown to induce release of WPBs [14]. In agreement with these data, we show that addition of S1P to a final concentration of 1 μM S1P results in a time-dependent increase of VWF levels in conditioned medium of endothelial cells (Figure 1A). Inspection of the increase in VWF levels suggests that following a rapid initial burst of VWF release that peaks at 10–15 minutes is followed by a more sustained rise in VWF levels that continues for at least 45 minutes (Figure 1A). S1P exerts its action through its binding to members of the family of G-protein coupled S1P receptors (S1PR). This receptor family consists of 5 members of which S1PR1 and S1PR3 have been shown to be expressed in endothelial cells [10]. Expression of S1PR1 and S1PR3 in human umbilical cord endothelial cells (HUVEC) was confirmed by qPCR (data not shown). To identify the receptor responsible for S1P-mediated signaling regulating VWF secretion, siRNA-mediated knockdown of S1PR1 and S1PR3 was performed. Specific down-regulation of S1P receptor 1 and 3 was observed (Figure 1B). Downregulation of S1PR1 expression did not significantly influence VWF secretion following stimulation with thrombin, forskolin or S1P (Figure 1C). A time-course revealed that also the kinetics of VWF release were not affected by down-regulation of endogenous S1PR1 (Figure 1D). However, downregulation of S1PR3 significantly reduced S1P-induced VWF secretion (Figure 1E). As expected, thrombin- and forskolin induced release of VWF was not affected by S1PR3 depletion (Figure 1E). Time-course experiments confirmed that S1PR3 depletion abolished S1P-induced release of VWF (Figure 1F).

**Figure 2:**
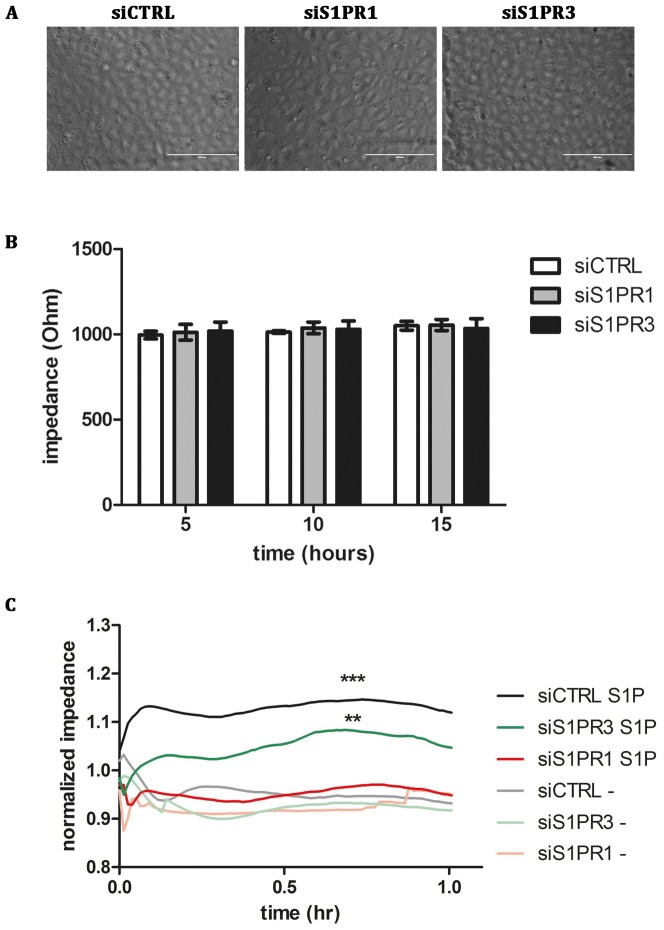
S1PR1 is responsible for barrier integrity in S1P stimulated HUVECs. (A) HUVECs were transfected with a control siRNA SMARTpool (siCTRL) or a siRNA SMARTpool targeting S1PR1 (siS1PR1) or S1PR3 (siS1PR3) and grown to confluence on electrode ECIS-arrays. (B) SiCTRL, siS1PR1 or siS1PR3 transfected HUVECs show no differences in impedance after the indicated time points. (C) HUVECs transfected with a control siRNA SMARTpool (siCTRL) or a siRNA SMARTpool targeting S1PR1 (siS1PR1) or S1PR3 (siS1PR3) were incubated with 1 μM S1P or SF medium alone. The impedance was continuously monitored and was corrected for starting impedance after 1 hr serum starvation. Three independent experiments were performed. Statistical significance was assessed by 2-way ANOVA followed by Bonferroni post-hoc test for selected comparison (***P<0.001; **P<0.01 and *P<0.05).

Treatment of endothelial cells with S1P has been described to improve endothelial barrier function[1]. S1P decreases vascular permeability and tightens cell-cell junctions due to Rac1 activation. SiRNA mediated down regulation of S1PR1 or S1PR3 did not affect endothelial monolayer formation (Figure 2A) or trans-endothelial cell resistance in non-stimulated cells (Figure 2B), as was assessed using ECIS. Upon incubation with S1P, an increase in trans-endothelial cell resistance was observed in agreement with previous findings (Figure 2C) [33]. SiRNA-mediated knockdown of S1PR3 expression did not prevent the S1P-induced increase in endothelial cell barrier function (Figure 2C). Conversely, knockdown of S1PR1 prevented the S1P-induced increase in endothelial cell barrier function (Figure 2C). These findings show that S1P-induced endothelial barrier function is mediated through S1PR1 but not S1PR3. Our findings suggest that S1PR1 and S1PR3 control different cellular processes.

### Pharmacological modulation of S1P-induced VWF release

**Figure 3:**
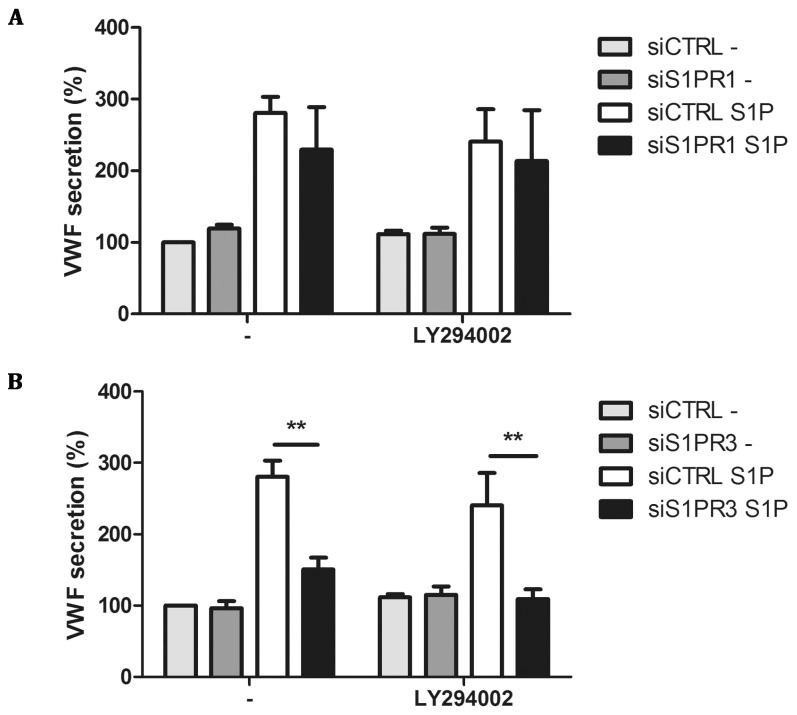
Inhibition of PI3 kinase does not affect S1P-induced WPB release. (A/B) HUVECs were transfected with a control siRNA SMARTpool (siCTRL) or a siRNA SMARTpool targeting S1PR1 (siS1PR1) or S1PR3 (siS1PR3). Transfected HUVECs were then incubated for 60 minutes with SF medium or SF medium containing 10 μM LY294002. After preincubation, HUVECs were stimulated for 45 minutes with 1 μM S1P, or SF medium alone (-) in presence or absence of PI3K inhibitor LY294002. The amount of VWF secreted in the medium was measured by ELISA. Total amount of VWF secreted is displayed in percentages; the amount of VWF secreted by unstimulated cells at 45 minutes in cells transfected with siCTRL was set to 100%. Three independent experiments were performed. Statistical significance was assessed by 2-way ANOVA followed by Bonferroni post-hoc test for selected comparison (***P<0.001; **P<0.01 and *P<0.05).

**Figure 4:**
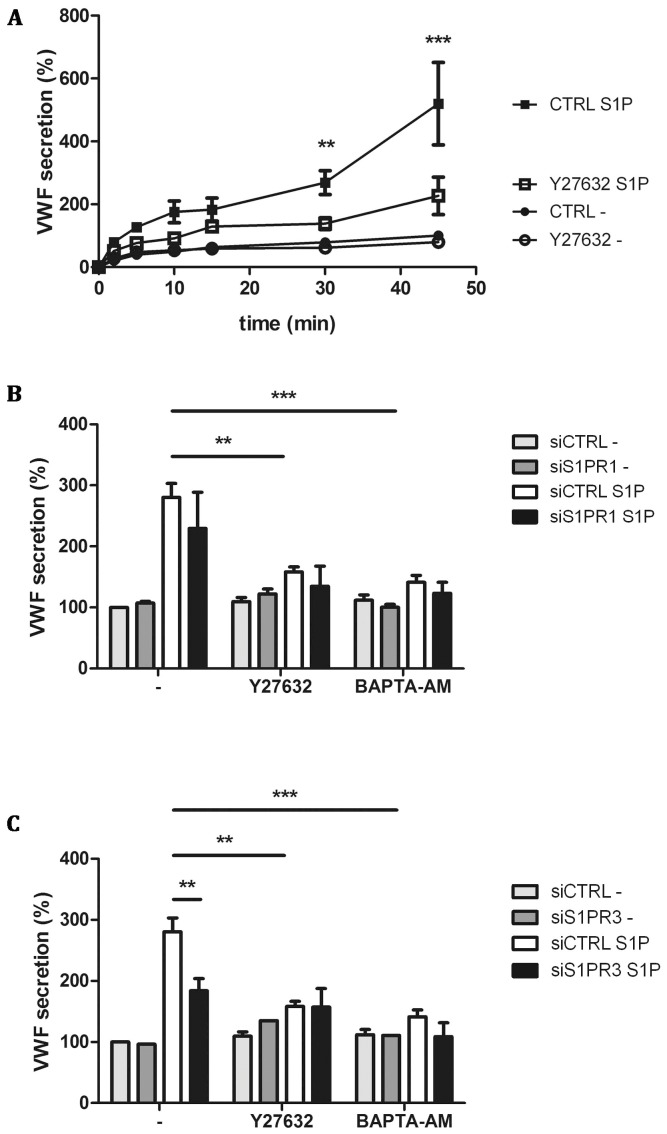
Pharmacological inhibition of S1P-induced VWF secretion. CTRL and Y27632 treated HUVECs were incubated for indicated times with 1 μM S1P or SF medium alone (-) (A). (B) siCTRL and siS1PR1 treated HUVECs were incubated for 45 minutes with 1 μM S1P or SF medium alone (-) in the presence of 10 μM Rho kinase inhibitor Y27632 or 100 μM calcium chelator BAPTA-AM. (C) siCTRL and siS1PR3 treated HUVECs were incubated for 45 minutes with 1 μM S1P or SF medium alone (-) in the presence of Rho kinase inhibitor Y27632 or calcium inhibitor BAPTA-AM. The amount of VWF secreted in the medium was measured by ELISA; VWF secreted by unstimulated siCTRL treated cells after 45 minutes was set to 100%. Three independent experiments were performed. Statistical significance was assessed by 2-way ANOVA followed by Bonferroni post-hoc test for selected comparison (***P<0.001; **P<0.01 and *P<0.05).

Binding of S1P to S1PR1 has been shown to activate G proteins belonging to the G_i_ family whereas S1PR3 signals through G_q_, G_i_ and G_12/13_ proteins [34]. G_i_, which can act downstream of both S1PR1 and S1PR3, has been shown to signal through phosphatidylinositide 3-kinase (PI3K) [35], [36]. Interestingly, pre-treatment of HUVECs with the PI3K inhibitor LY294002 did not prohibit S1P-induced release of VWF (Figure 3A). We also addressed whether LY294002 inhibited VWF release in cells depleted for S1PR1 and S1PR3. Also, under these conditions LY294002 did not affect the S1P-induced release of VWF (Figure 3A and B). S1PR3 mediated G_q_ dependent signaling has been described to upregulate intracellular calcium in a PLCγ dependent manner, while G_12/13_ has been described to activate Rho and Rho-associated protein kinase (ROCK) [37], [38]. We addressed whether S1P-induced release of VWF can be blocked by the ROCK inhibitor Y27632. Pre-incubation of HUVECs with Y27632 greatly reduced time-dependent S1P-induced release of VWF (Figure 4A). S1PR3-mediated G_q_ dependent signaling has been described to upregulate intracellular calcium in a PLCγ dependent manner [39]. To assess the involvement of G_q_ -mediated rise in intracellular Ca^2+^ in the release of VWF, we preincubated cells with the calcium chelator BAPTA-AM. S1P-induced release of VWF was completely abolished in the presence of BAPTA-AM (Figure 4B). In accordance with previous data, thrombin-induced release of VWF was also significantly decreased in the presence of BAPTA-AM whereas epinephrine-induced release of VWF was not affected (data not shown). Therefore, we also determined the effect of Y27632 and BAPTA-AM on S1P-induced release of VWF in S1PR1- and S1PR3-depleted cells. Both Y27632 and BAPTA-AM reduced S1P-induced release of VWF in S1PR 1 depleted cells (Figure 4B). BAPTA-AM and Y27632 also further attenuated the observed inhibition of S1P-induced VWF release in S1PR3 depleted cells (Figure 4C). These results suggest that interfering with downstream targets of G_q_ and G_12/13_ abolishes S1P-induced release of VWF. Altogether the pharmacological profile observed for S1P induced release of VWF is consistent with a role for S1PR3 in triggering the release of WPBs from endothelial cells.

## Discussion

In this study we show that S1PR3 mediates S1P-induced Weibel-Palade body exocytosis in human endothelial cells. Our results extend previous findings showing that S1P stimulation induces WPB release in a calcium dependent manner [14]. At high concentrations of LY294002, potentiation of VWF release by S1P was observed [14]. Based on these findings it was proposed that PI3K-dependent activation of eNOS, leading to the generation of nitric oxide, resulted in the inhibition of WPB release. In contrast, we did not observe potentiation of S1P-induced VWF release by inhibition of PI3K (Figure 3). One explanation may be the use of different endothelial cell types. Matsushita and co-workers used human aortic endothelial cells whereas we used human umbilical vein-derived endothelial cells for our studies. Differences in expression levels of eNOS between endothelial cells derived from different vascular beds may explain the observed differences between the two studies [40].

Using siRNA depletion we show that the S1PR1 is not involved in S1P-induced release of VWF (Figure 1). The S1PR1 is coupled to Gα proteins of the G_i_ subfamily [34]. Interestingly, Matsushita and co-workers reported that S1P induced release of VWF was blocked by pertussin toxin which blocks G protein coupled receptor signaling through members of the G_i_ subfamily [14]. We did not use this compound in our studies but the lack of inhibition of the PI3K inhibitor LY294002 observed in this study suggests that G_i_ -mediated signaling through PI3K is not crucially involved in S1P-induced WPB release.

**Figure 5:**
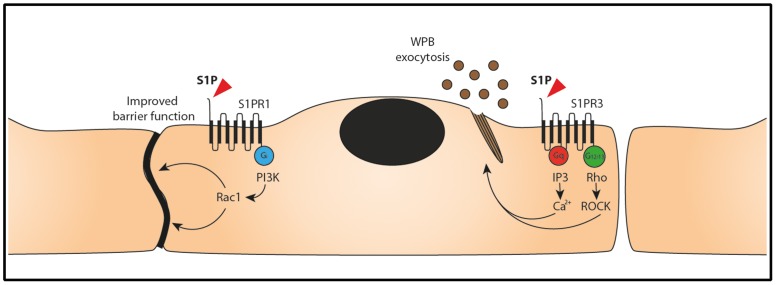
Signaling pathways that regulate S1P induced WPB exocytosis. Stimulation of the S1P receptor 1 (S1PR1) by S1P induces G_i_ subunit mediated Rac1 activation by PI3K- dependent signaling, resulting in increased barrier function. Stimulation of S1PR3 by S1P results in activation of G_q_ and G_12/13_ subunits. G_q_ subunit activation results in an increase in intracellular calcium levels. Activation of G_12/13_ subunits signals to RhoA and ROCK that promote assembly of actin rings that facilitate WPB exocytosis [48].

A large number of studies have firmly documented that S1P enhances endothelial cell barrier function [1], [9]–[12], [41]. S1P mediates this effect on endothelial cell barrier function through S1P receptor 1 [12]. In this study we confirmed that the effects of S1P on endothelial cell barrier function are dependent on S1PR1 and not S1PR3 (Figure 1). S1P has been shown to have a beneficial effect on endothelial cell barrier function in animal models of acute lung injury (ALI) [41]. A signaling pathway downstream of S1PR1 involving the small GTPase Rac1 has been shown to contribute to barrier-enhancement [12], [41]. Interestingly, signaling through S1PR3 has been proposed to decrease barrier function [41] which may account for the pleiotropic effect of S1P administration in animal models of ALI. Under our experimental conditions we did not observe a decrease in barrier function in endothelial cell depleted for S1PR1 (Figure 2). Overall, our findings are consistent with a functional dichotomy of S1P receptors 1 and 3. S1PR1 controls endothelial cell barrier function whereas binding of S1P to S1PR3 induces release of WPBs (Figure 5). At present, we do not know how these processes are regulated. It is well-established that a S1P gradient regulates S1PR1-dependent trafficking of lymphocytes from secondary lymphoid organs to the blood circulation [42]. This S1P-gradient is also utilized for the S1PR1-dependent formation of platelets from megakaryocytic pro-platelet strings in the blood stream [43]. Our results show that S1P-induced release of inflammatory, pro-angiogenic and hemostatic cargo from endothelial cell-specific WPBs is dependent on S1PR3. We speculate that under quiescent conditions circulating levels of S1P interact primarily with S1PR1 thereby ensuring maintenance of endothelial cell barrier function as well as platelet and lymphocyte recruitment to the blood circulation. The S1P-concentration in blood varies between 0.4 and 1.5 μM with most of the S1P being bound to lipoproteins in plasma [44], [45]. Both erythrocytes and platelets have been shown as a major source of S1P [6], [46].

We propose that following activation of platelets and/or fragmentation of red blood cells due to vascular obstruction massive amounts of S1P become available that induce release of WPBs through its binding to S1PR3. The hemostatic and angiogenic components present in these organelles will contribute to restoration of vascular homeostasis which may include neovessel formation. Exposure of P-selectin and release of other inflammatory components present in WPBs may contribute to local recruitment of neutrophils. In this respect it is interesting to note that high concentrations of S1P have been shown to disrupt barrier function through binding to the S1PR3 [47]. These data suggest that high levels of S1P may promote barrier-dysfunction by overcoming a critical threshold for RhoA activation that overcomes the barrier-protective Rac1 mediated signaling via S1P receptor 1 [41]. Future studies need to show whether the pleiotropic effects of S1P administration can be explained through this mechanism. In this study we document that barrier-protective concentrations of S1P induces release of WPBs through S1PR3.
